# ﻿Description of the first species of *Scutigerella* (Symphyla, Scutigerellidae) from China, with mitogenomic and genetic divergence analysis

**DOI:** 10.3897/zookeys.1157.99686

**Published:** 2023-04-05

**Authors:** Ya-Li Jin, Nerivania Nunes Godeiro, Yun Bu

**Affiliations:** 1 Shanghai Natural History Museum, Shanghai Science & Technology Museum, Shanghai, 200041, China Shanghai Natural History Museum Shanghai China

**Keywords:** DNA barcode, genetic distance, mitochondrial genome, Myriapoda, symphylans, taxonomy

## Abstract

*Scutigerellasinensis* Jin & Bu, **sp. nov.** from China is described and illustrated. It is characterized by a deeply emarginated posterior margin of tergite 2, less differentiated marginal setae on all tergites, absence of seta a3 around the antennal base, and 6–8 setae on the first tergite. The complete mitochondrial genome of the new species is also analyzed and compared with the mitogenome of *Scutigerellacauseyae*. In the reconstructed Neighbor-Joining tree based on COI gene sequences, *S.sinensis***sp. nov.** clusters with *S.causeyae*, however, with big distances. The genetic divergence among *S.sinensis***sp. nov.** and congeners, species of *Hanseniella* and *Scutigerella*, and both families of Symphyla was analyzed using COI gene sequences.

## ﻿Introduction

The class Symphyla Ryder, 1880 is a monophyletic group of myriapods with worldwide distribution (Szucsich and Scheller 2011). However, both taxonomic and molecular studies are widely missing. There are only about 250 species reported worldwide (https://www.itis.gov/; Jin and Bu 2020, 2023) and ten species recorded in China until now (Bu and Jin 2018; Jin and Bu 2018, 2019, 2020, 2023; Jin et al. 2019). Only 65 mitochondrial gene sequences and two complete mitogenomes are available in GenBank (accessed in February 2023). The sub-cosmopolitan genus *Scutigerella* Ryder, 1882 is the second largest group of the family Scutigerellidae. It includes 36 valid species registered in the Integrated Taxonomic Information System (ITIS; https://www.itis.gov/) (accessed in February 2023). However, *Scutigerellagratiae* (Ryder, 1881) is missing in the database of ITIS, but it is recorded in the virtual research environment Myriatrix, The Fellegship of the Rings (2020 onwards) (http://myriatrix.myspecies.info), thus 37 valid *Scutigerella* species in total. The occurrence of *Scutigerella* in China (Hunan Province) was only once mentioned in a monograph (Zhang and Wang 1992), but the species remained undetermined.

In recent years, many specimens of Scutigerellidae Bagnall, 1913 were obtained from Shanghai and Beijing and were carefully studied, and most of them belong to the genus *Hanseniella* Bagnall, 1913. Among those specimens, one species of *Scutigerella* was identified as new to science and is described in the present paper. It is also the first species of the genus from China. In order to provide further evidence for the new species and clarify its taxonomic position, its complete mitogenome was sequenced and analyzed. In addition, the phylogenetic relationship and genetic divergence of symphylans were analyzed based on DNA barcode sequences.

## ﻿Materials and methods

### ﻿Sample collection and taxonomy

Soil and litter samples from broad-leaf and bamboo forests from Dajinshan Island, Shanghai were collected during several ecological surveys of soil fauna between 2015–2018, and specimens were extracted using Berlese-Tullgren funnels and preserved in 80% ethanol. Materials from Beijing were collected in Yuan-Ming Yuan Imperial Garden by Mr Rui-Qing Wang in 2021. They were mounted on slides using Hoyer’s solution and dried in an oven at 50 °C. Morphological observations were performed under a phase contrast microscope (Leica DM 2500). Photographs were taken with a digital camera installed on the microscope (Leica DMC 4500). Line drawings were done using a drawing tube. All specimens are deposited in the collections of the Shanghai Natural History Museum (**SNHM**), Shanghai, China.

### ﻿Molecular analyses

The specimens used for the experiment were collected by Ya-Li Jin and Yun Bu from Dajinshan Island on 11 November, 2017. Samples were preserved in absolute ethanol at -20 °C for DNA extraction. Prior to DNA extraction, a single individual was mounted on a temporary slide using absolute ethanol to confirm the species identification. One specimen, preserved in alcohol, was sent to Shanghai Yaoen Biotechnology Co., Ltd, China, where all laboratory procedures, including DNA extraction and library construction were made following custom procedures. DNA was extracted from a single individual of the species using the TIANamp MicroDNA extraction kit (Tiangen Co., Ltd, China). Libraries were constructed using KAPA Hyper Prep Kit (Roche). An Illumina NovaSeq platform was used to produce paired-end reads with 150 bp length. Approximately 10 Gb of data from the species was generated and used to assemble the mitogenomes.

### ﻿Sequence analysis

NovoPlasty v.3.8.3 (Dierckxsens et al. 2016) was used to assemble the mitogenome using the COI sequence from *Scutigerellacauseyae* Michelbacher, 1942 retrieved from GenBank as a seed (accession number NC008453). Genes annotation was performed using MitoZ ﻿v.2.4-alpha (Meng et al. 2019). The final mitogenome sequence with annotations and the raw sequencing data were submitted to the National Center for Biotechnology Information database (NCBI), accession numbers are listed in Table 1. The mitogenomic data of *Scutigerellacauseyae* were downloaded from GenBank (https://www.ncbi.nlm.nih.gov/), and the length, genes arrangement, nucleotides content, and other genomic features were compared with *Scutigerellasinensis* sp. nov.

**Table 1.** Taxonomical and collection information of the species used in the analysis.

Species and voucher	Family	Genus	Country	GenBank number	Reference
*Scutigerellasinensis* sp. nov. JYL-DJS2017011	Scutigerellidae	*Scutigerella*	China	OQ165321	Present study
*Scutigerellacauseyae*	Scutigerellidae	*Scutigerella*	Germany	NC008453	Podsiadlowski et al. 2007
*Scutigerella* sp. WAMT144261	Scutigerellidae	*Scutigerella*	Australia	MW021294	Cullen and Harvey 2020 (unpublished)
*Scutigerella* sp. WAMT144298	Scutigerellidae	*Scutigerella*	Australia	MW021295	Cullen and Harvey 2020 (unpublished)
*Scutigerella* sp. WAMT145461	Scutigerellidae	*Scutigerella*	Australia	MW021296	Cullen and Harvey 2020 (unpublished)
*Scutigerella* sp. WAMT145462	Scutigerellidae	*Scutigerella*	Australia	MW021297	Cullen and Harvey 2020 (unpublished)
*Scutigerella* sp. WAMT145463	Scutigerellidae	*Scutigerella*	Australia	MW021298	Cullen and Harvey 2020 (unpublished)
*Scutigerella* sp. PU234	Scutigerellidae	*Scutigerella*	Australia	MT457863	Cullen and Harvey 2020 (unpublished)
*Hanseniella* sp. BMR00202	Scutigerellidae	*Hanseniella*	Australia	MT902530	Gunawardene et al. 2020 (unpublished)
*Hanseniella* sp. BMR00229	Scutigerellidae	*Hanseniella*	Australia	MT902546	Gunawardene et al. 2020 (unpublished)
*Hanseniella* sp. BMR00230	Scutigerellidae	*Hanseniella*	Australia	MT902547	Gunawardene et al. 2020 (unpublished)
*Hanseniella* sp. BMR00231	Scutigerellidae	*Hanseniella*	Australia	MT902548	Gunawardene et al. 2020 (unpublished)
*Hanseniella* sp. BMR00232	Scutigerellidae	*Hanseniella*	Australia	MT902549	Gunawardene et al. 2020 (unpublished)
*Hanseniella* sp. BMR00243	Scutigerellidae	*Hanseniella*	Australia	MT902557	Gunawardene et al. 2020 (unpublished)
*Hanseniella* sp. BMR00364	Scutigerellidae	*Hanseniella*	Australia	MT902595	Gunawardene et al. 2020 (unpublished)
*Hanseniella* sp. BMR01208	Scutigerellidae	*Hanseniella*	Australia	MT902776	Gunawardene et al. 2020 (unpublished)
Scutigerellidae sp. FRL-2015	Scutigerellidae	undetermined	Colombia	KP696390	Salazar-Moncada et al. 2015
Scutigerellidae sp. BMR00070	Scutigerellidae	undetermined	Australia	MT902426	Gunawardene et al. 2020 (unpublished)
Scutigerellidae sp. BMR00071	Scutigerellidae	undetermined	Australia	MT902427	Gunawardene et al. 2020 (unpublished)
Scutigerellidae sp. BMR00241	Scutigerellidae	undetermined	Australia	MT902555	Gunawardene et al. 2020 (unpublished)
Scutigerellidae sp. BMR00242	Scutigerellidae	undetermined	Australia	MT902556	Gunawardene et al. 2020 (unpublished)
Scutigerellidae sp. BMR00244	Scutigerellidae	undetermined	Australia	MT902558	Gunawardene et al. 2020 (unpublished)
Scutigerellidae sp. BMR00641	Scutigerellidae	undetermined	Australia	MT902704	Gunawardene et al. 2020 (unpublished)
Scutigerellidae sp. BMR01199	Scutigerellidae	undetermined	Australia	MT902772	Gunawardene et al. 2020 (unpublished)
Scutigerellidae sp. BMR01576	Scutigerellidae	undetermined	Australia	MT621062	Gunawardene et al. 2020 (unpublished)
Scutigerellidae sp. BMR01578	Scutigerellidae	undetermined	Australia	MT621064	Huey and Floeckner 2020 (unpublished)
Scutigerellidae sp. BMR01587	Scutigerellidae	undetermined	Australia	MT621072	Huey and Floeckner 2020 (unpublished)
*Symphylella* sp. YG-2006	Scolopendrellidae	Symphylella	China	NC011572	Gai et al. 2008

In order to make a comprehensive analysis of genetic divergences among symphylans, DNA barcode sequences (COI gene, 658 base pairs) of 26 sequences of the family Scutigerellidae and one sequence of the family Scolopendrellidae Newport, 1844 (outgroup) were downloaded from GenBank and analyzed. The detailed information and accession numbers of the 28 sequences analyzed in this study are listed in Table 1. To infer the position of the new species described, the Neighbor-Joining tree was constructed based on COI gene sequences by MEGA X (Kumar et al. 2018) with the Jukes-Cantor model (Jukes and Cantor 1969) and 1000 bootstrap replicates. The genetic distance (K2P-distance) was calculated using MEGA X (Kimura 1980; Kumar et al. 2018) and the genetic divergence was analyzed for different taxonomic levels of Symphyla.

### ﻿Data availability statement

After publication, mitogenome sequence and raw sequencing data will be available in GenBank (NCBI) at https://www.ncbi.nlm.nih.gov/ under the accession numbers OQ165321/PRJNA900014.

## ﻿Results

### ﻿Taxonomy

**Class Symphyla Ryder, 1880**

**Family Scutigerellidae Bagnall, 1913**

#### ﻿Genus Scutigerella

Taxon classificationAnimaliaScutigeromorphaScutigerellidae

Ryder, 1882

114F2044-4D24-5B1A-BC60-FF3AD55EBB2D

##### Type species.

*Scolopendrellaimmaculata* Newport, 1845. Valid name: *Scutigerellaimmaculata* (Newport, 1845).

##### Diagnosis.

Trunk with 15 tergites. Four macrosetae (a1–a4) around the antennal base, rarely seta a3 absent. Posterior margins of tergites emarginated. Last tergite with a deep cavity between cerci. First pair of legs with 4 segments, others with 5 segments. Styli present at the base of legs 3–12. Coxal sacs present at the base of legs 3–10.

##### Distribution.

Sub-cosmopolitan (Szucsich and Scheller 2011).

#### ﻿ Scutigerella sinensis

Taxon classificationAnimaliaScutigeromorphaScutigerellidae

Jin & Bu sp. nov.

7DB76F7C-8E2C-5342-AA03-7C3640EABC8D

https://zoobank.org/21204C04-5009-40AE-B37F-2DC2532C62F3

**Figure 1.**
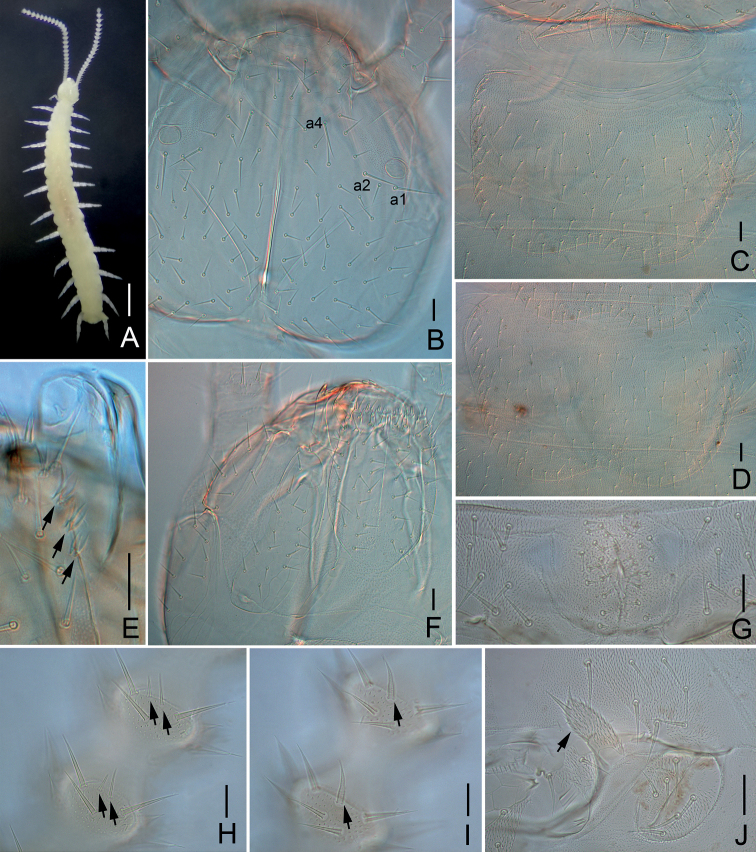
*Scutigerellasinensis* Jin & Bu, sp. nov. **A** habitus, dorsal view in alcohol **B** head, dorsal view (a1, a2 and a4–macrosetae around antennal base) **C** tergites 1–2 **D** tergite 3 **E** first maxilla and the right part of second maxilla (arrows indicate spined organs) **F** head, ventral view **G** male genitalia **H** right 10–11 antennomeres, dorsal view (arrows indicate spiniform sensory organs) **I** right 13–14 antennomeres, ventral view (arrows indicate sensory setae) **J** stylus and coxal sac on base of leg 5 (arrow indicates stylus). Scale bars: 500 μm (**A**); 20 μm (**B–J**).

**Figure 2.**
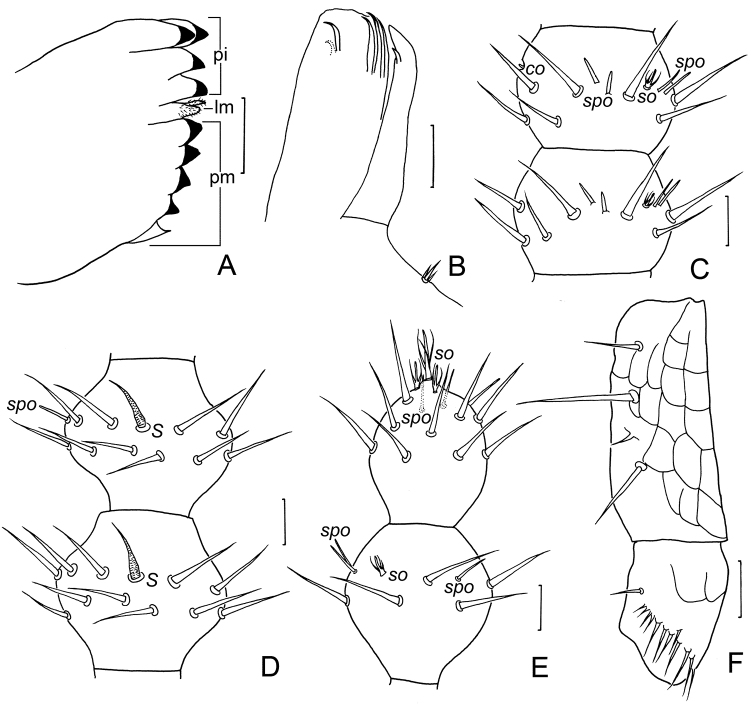
*Scutigerellasinensis* Jin & Bu, sp. nov. **A** mandible, lateral view (*pi*–pars incisivus, *pm*–pars molaris, *lm*–lacinia mobilis) **B** first maxilla **C** right 6–7 antennomeres, dorsal view (*co*–conical sensory organ, *spo*–spiniform sensory organs, *so*–spined sensory organs) **D** right 13–14 antennomeres, ventral view (*S*–sensory setae) **E** terminal two antennomeres, dorsal view **F** femur and tibia of leg 1, ventral view. Scale bars: 20 μm.

**Figure 3.**
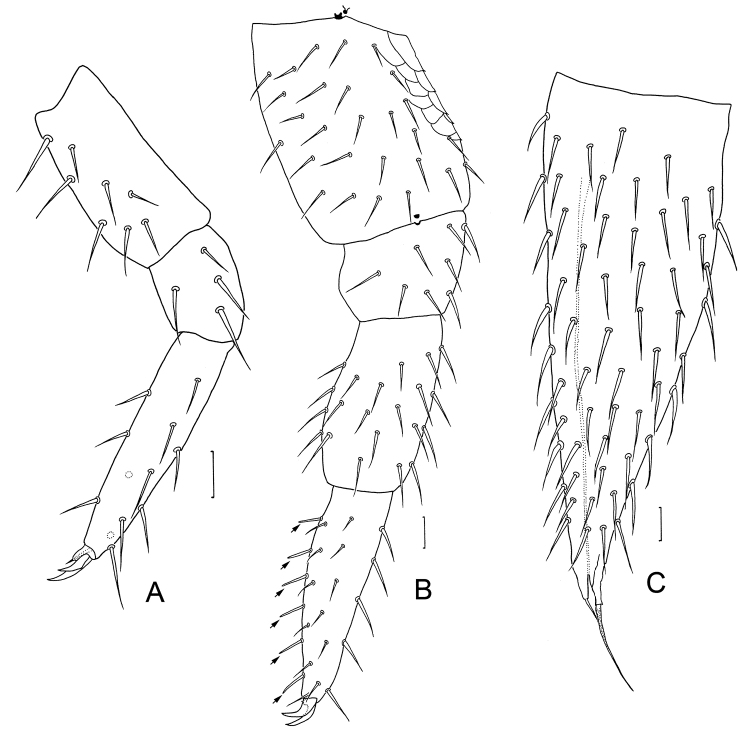
*Scutigerellasinensis* Jin & Bu, sp. nov. **A** leg 1, dorsal view **B** leg 12, ventro-lateral view (arrows indicate spiniform setae) **C** left cercus, dorsal view. Scale bars: 20 μm.

Figs 1 , 2 , 3 , Table 2

##### Diagnosis.

*Scutigerellasinensis* sp. nov. is characterized by absence of a3 seta around the antennal base, 6–8 setae on the first tergite, deeply emarginated posterior margin of tergite 2, 28–37 marginal and 41–57 inner setae on tergite 2, less differentiated marginal setae on all tergites, femur of first pair of legs without a conspicuous ventral process, posterior styli without a lateral seta, cavity of fifteenth tergite V-shaped, tarsus of last pair of legs moderately set with setae, cerci 2.7–3.4 times as long as width, cerci densely covered with subequal setae, cerci without expansion in terminal area.

##### Material examined.

***Holotype***: male (slide no. SH-DJS-SY2015009) (SNHM), China, Shanghai, Dajinshan Island, extracted from soil samples of bamboo forest, alt. 103 m, 30°41'N, 121°26'E, 30-VI-2015, coll. Y. Bu & Y. L. Jin. ***Paratypes***: 1 female (slide no. SH-DJS-SY2017001), ibidem, 11-XI-2017; 1 female (slide no. SH-DJS-SY2017002), ibidem, extracted from soil samples of broad-leaf forest, 11-XI-2017, coll. Y. Bu & Y. L. Jin; 1 female (slide no. SH-DJS-SY2018003), ibidem, 24-IV-2018, coll. Y. Bu & J. Y. Li; 1 male (slide no. SH-DJS-SY2018001), ibidem, extracted from soil samples of broad-leaf forest, 24-X-2018, coll. Y. Bu & J. Y. Li; 1 female (slide no. BJ-YMY-SY2021001), China, Beijing, Yuan-Ming Yuan Imperial Garden, extracted from soil samples of a deserted field with herbaceous plants, alt. 60 m, 40°1'N, 116°17'E, 15-IV-2021, coll. R. Q. Wang. ***Non-type specimens***: 1 juvenile with 8 pairs of legs (slide no. SH-DJS-SY2015002), same data as holotype; 1 juvenile with 9 pairs of legs (slide no. SH-DJS-SY2015111), ibidem, 22-IX-2015, coll. Y. Bu & Y. L. Jin; 1 juvenile with 11 pairs of legs (slide no. SH-DJS-SY2018002), ibidem, 24-IV-2018, coll. Y. Bu.

##### Description.

Adult body 3.4 mm long on average (3.0–4.3 mm, *N* = 6), holotype 3.2 mm (Fig. 1A).

***Head*** length 300–350 μm, width 320–420 μm, broadest part just posterior of midlength, dorsally covered with straight setae of varying lengths (Fig. 1B). Antennal base with 3 macrosetae: a1, a2 and a4 (33–50 μm), a3 absent (Fig. 1B). Longest seta (43–53 μm) located between Tömösváry organ and spiracle, same length with greatest width of third antennomere. Reticulation of cuticular thickenings present on frons. Central rod complete (150–187 μm), less distinct at most anterior portion, 0.5 times the length of head, with distinct ovoid swollen end (Fig. 1B). Dorsal cuticle of head glabrous.

***Tömösváry organ*** subspherical, length 20–22 μm, width 15–20 μm, 0.3–0.4 times as wide as greatest diameter of third antennomere (Fig. 1B).

***Mouthparts*.** Mandible similar to *Hanseniella*. Pars incisivus with four distinct thick teeth, pars molaris with four smaller teeth and one proximal spine, lacinia mobilis with 2 pubescent processes observed from lateral view under light microscope (Fig. 2A). First maxilla has two lobes, inner lobe with 4 hook-shaped dorsal teeth and 1 tiny ventral tooth, palp small, with three pointed branches, middle one distinctly longer than lateral ones (Fig. 2B). Second maxilla with many small protuberances anteriorly, each carrying one seta, distal setae thicker and spiniform, posterior part with sparse setae, 3+3 spined organs present on anterolateral margin (Fig. 1E, F). Cuticle of second maxilla covered with dense pubescence.

***Antennae*** with 19–29 antennomeres (holotype with 29), about 0.4 of body length. First antennomere cylindrical, 1.3–2.5 times wider than long (length 30–45 μm, width 50–75μm), with 5–6 setae in one whorl, longest seta 28–30 μm, about half of antennomere width. Second antennomere 1.3–1.9 times wider than long (width 48–65 μm, length 33–40 μm), with 7–9 setae evenly inserted, longest seta inserted outer-dorsally, about 0.5–0.7 times as long as antennomere width. Third antennomere 1.3–1.9 times wider than long (length 25–40 μm, width 45–65μm), with primary whorl of 7–10 setae, longest seta 0.5–0.7 times as long as antennomere width. Setae on proximal antennomeres longer and on distal antennomeres shorter. Proximal antennomeres each with only primary whorl of setae. Secondary whorl setae appear from antennomeres 6–8 to penultimate antennomere (Figs 1H, I, 2C, D). Four kinds of sensory organs observed on antenna: spiniform sensory organs present on antennomeres 3–5 to distal antennomere increasing in number from 2 to 8, short and thick on proximal antennomeres (Fig. 2C, D), long and slender on distal ones (Figs 1H, 2E); one small spined sensory organ consists of several spine and central stub present on dorsal side from fourth antennomere onwards to subdistal antennomere, rarely absent (Fig. 2C, E); single tiny conical sensory organ present on outer side of several antennomeres discontinuously distributed along the antenna (Fig. 2C); one huge spined sensory organ only present on distal antennomere, distinctly bigger than small ones (Fig. 2E). Additionally, one sensory seta decorated with transverse stripes always present on ventral side from second antennomere onwards to penultimate antennomere (Figs 1I, 2D). Distal antennomere longer than wide (length 58–70 μm, width 50–58 μm), with 1 huge spined sensory organ consisting of three or four curved spines stemming from one central stalk on elevated base about 0.3–0.4 times as long as width of antennomere and two neighboring medium ones, 4–7 spiniform organs and 15–22 normal setae on distal half (Fig. 2E). Cuticular reticulation present on first and second antennomere, mainly transverse. All antennomeres pubescent. Chaetotaxy and sensory organs of antennae of holotype are given in Table 2.

**Table 2.** Numbers of normal setae and sensory organs on antennae of *Scutigerellasinensis* Jin & Bu sp. nov. (holotype).

Antennomere	Normal setae	Spiniform sensory organs	Spined sensory organs	Conical sensory organs	Ventral sensory setae
1	5				
2	9				1
3	10				1
4	10	1	1		1
5	11	3	1		1
6	14	4	1		1
7	17	4	1	1	1
8	18	4	1		1
9	19	4	1	1	1
10	19	4	1		1
11	20	4	1	1	1
12	20	4	1		1
13	20	5	1		1
14	20	4	1		1
15	19	6	1	1	1
16	18	6	1		1
17	17	5	1		1
18	17	5	1		1
19	17	5	1	1	1
20	18	5	1		1
21	17	6	1	1	1
22	16	5	0		1
23	17	6	1		1
24	17	6	1		1
25	18	6	1	1	1
26	18	6	0		1
27	14	8	1	1	1
28	16	5	1		1
29	22	7	3		

***Tergites*.** Tergite 1 rudimentary, with 6–8 subequal setae in one row (Fig. 1C). Tergite 2 complete, 1.6 times wider than long (width 295–245 μm, length 155–185 μm), posterior margin deeply emarginated, with 28–37 subequal marginal setae, longest one (25–27 μm) 1.7–1.8 times as long as shortest one (15–17 μm), 0.4–0.6 and 0.2 times as third antennomere respectively; areas surrounded by marginal setae covered by 41–57 inner setae, similar to marginal setae; anterior half with short pubescence on mesh-work covered cuticular thickenings, posterior half with fine dense pubescence (Fig. 1C). Tergite 3 complete, broader and longer than tergite 2, 1.5–1.6 times wider than long (width 305–382 μm, length 190–240 μm), posterior margin deeply emarginated, with 36–42 subequal marginal setae, longest one (22–29 μm) 1.7–1.8 times as long as shortest one (12–17 μm), with 58–89 subequal inner setae (Fig. 1D). Tergite 4 broader than tergite 3, with 32–39 subequal marginal setae and 44–66 subequal middle setae. Shape of tergites 5–7, 8–10, and 11–13 similar as tergites 2–4. Pattern of alternating tergite lengths of two short-tergites followed by one long-tergite but disrupted at tergite 13. Last tergite with a V-shaped cavity located medially on posterior border. Anterolateral setae on all tergites not differentiated. Cuticle of all tergites densely pubescent. Posterior border of tergites glabrous (Fig. 1C, D).

***Legs*.** First pair of legs with 4 segments, trochanter absent; femur 1.8–2.0 times longer than wide (length 38–85 μm, width 28–43 μm), with cuticular reticulation (Fig. 2F), with 9–11 setae (Fig. 3A), longest seta (33–43 μm) 0.8–1.1 times as long as greatest width of femur; tibia 1.1–1.4 times as long as wide (40–50 μm, 30–38 μm), with total 4 or 5 setae, long pectinate setation (tibial pecten) present distolaterally (Fig. 2F), dorsal longest seta (23–28 μm) about 0.6–0.9 times as long as greatest diameter of tibia; tarsus about 3.3–4.6 times as long as wide (65–115 μm, 20–25 μm), slowly tapering towards distal end, with 8–14 setae, longest dorsal setae (18–25 μm) 0.8–1.0 times as long as greatest width of tarsus (Fig. 3A). Two slightly curved claws, anterior one somewhat broader and longer than posterior one (Fig. 3A). Leg 12 with 5 segments, 1.0–1.6 times as long as length of head; trochanter 1.4–1.7 times as long as wide (113–150 μm, 75–95 μm), dorsal side with cuticular reticulation, with 11–25 setae in total, longest one (20–21 μm) 0.2–0.3 times of greatest width of podomere (Fig. 3B); femur 1.1–1.3 times as long as wide (55–95 μm, 58–75 μm), with 6–14 setae and dorsal longest seta (23–28 μm) about 0.3–0.5 times as long as width of podomere (Fig. 3B); tibia nearly 1.5–1.9 times longer than wide (80–125 μm, 55–68 μm), with 5 or 6 longitudinal rows of setae, each row with 2–5 setae, longest outer seta (23–30 μm) 0.2–0.3 times as long as greatest width of podomere (Fig. 3B); tarsus 2.9–4.0 times as long as wide (105–150 μm, 38–43 μm) with 5 or 6 longitudinal rows of setae, each row with 2–6 setae, outer rows of setae straight and protruding, other setae slightly curved and short, longest seta (23–25 μm) 0.6–0.7 times as long as greatest width of podomere, 4–6 spiniform setae in a row present on ventral surface (Fig. 3B). Two claws slightly curved, almost same size. All legs covered with dense pubescence except areas with cuticular reticulation.

***Coxal sacs*** present at bases of legs 3–10, fully developed, each with 5 or 6 setae on surface (Fig. 1J). Corresponding area of leg 1, 2, 11, and 12 replaced by 2–3, 2–6, 2–3 and 1 seta respectively.

***Styli*** present at base of legs 3–12, 2.6–4.3 times as long as wide (33–58 μm, 10–20 μm), pubescent, with two distal setae, subapical seta (15–23 μm) 0.3–0.5 times as long as stylus, apical seta (8–10 μm) 0.2–0.3 times as long as stylus, both with pointed apex (Fig. 1J).

***Sense calicles*** located on two ventral protuberances of last tergite, posterior to base of leg 12, with smooth margin around pit. Sensory seta inserted in cup center, extremely long (235–275 μm).

***Cerci*** about 0.7–0.9 of head length, distinctly shorter than leg 12, 2.7–3.4 times as long as its greatest width (240–300 μm, 80–110 μm), moderately covered with subequal setae (Fig. 3C). Dorsally with 30–59 setae, ventrally with 26–57 setae; longest seta (28–33 μm) 1.3–1.5 times as long as shortest seta (20–28 μm), 0.3–0.4 times as long as greatest width of cercus and 0.1 times as length of cercus. Terminal area glabrous, 0.1 times as long as cercus. Two apical setae pointed, longer seta (43–58 μm) 0.2 times as long as cercus, with granules; shorter seta about a third of the length of longer one. Cuticle with dense pubescence.

***Male genitalia*** with 30 setae in total in holotype, without specialized setae (Fig. 1G).

##### Etymology.

This new species is named after the country of origin, from the Latin adjective sinensis, meaning Chinese.

##### Distribution.

China (Shanghai, Beijing).

##### Ecology.

Our current investigation indicates that *Scutigerellasinensis* sp. nov. is a rare species in natural habitats with very low density. We found about ten individuals among several hundred symphylans from plots of different vegetation. This *Scutigerella* is often coexisting with other dominant species of *Hanseniella* and *Symphylella* in the upper soil layer (0–10 cm) or humus.

##### Remarks.

The head chaetotaxy was briefly described in the previous studies of *Scutigerella*, usually with only shapes and numbers mentioned. The macrosetae around the base of the antenna of *Scutigerella* have been noticed and named by former researchers, and all species examined until now have a complete set of four macrosetae (a1–a4), which was deemed as a good diagnostic character in the taxonomy of *Scutigerella* (Hinschberger 1950; Juberthie-Jupeau and Tabacaru 1968; Scheller 1986). *Scutigerellasinensis* sp. nov. has three macrosetae (a1, a2, and a4) around the antennal base, with a3 seta absent, which can be easily distinguished from all other congeners. Our observation indicates this character is stable in both adults and juveniles and can be a unique feature of the new species. The present new species is most similar to the cosmopolitan species *Scutigerellaimmaculata* (Newport, 1845) in the shapes and chaetotaxy of tergites and legs, but differs in the absence of the a3 seta on the head (present in *S.immaculata*), number of marginal setae on tergite 2 and 3 (less than 50 in *S.sinensis* sp. nov. vs. more than 50 in *S.immaculata*), and the shape of the stylus (tapering in *S.sinensis* sp. nov. vs. cylindrical in *S.immaculata*).

### ﻿Mitogenomic analysis

**Figure 4.**
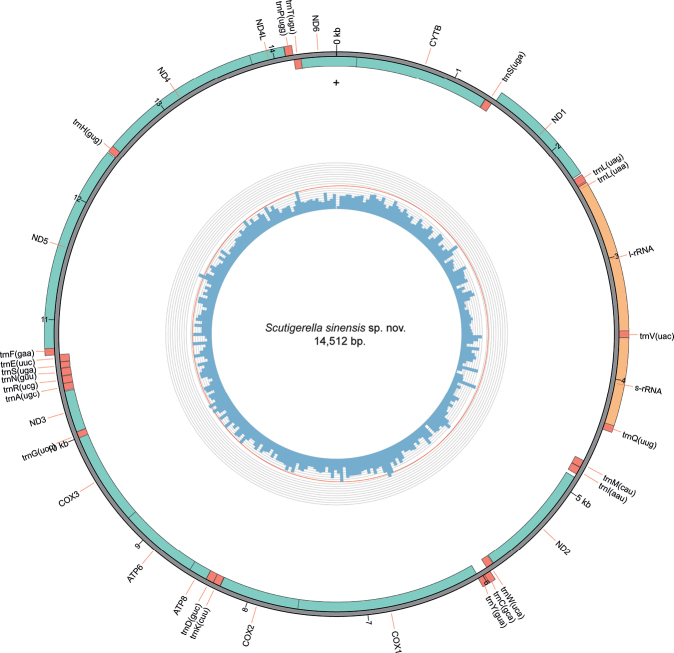
Circular representation of the mitogenome of *Scutigerellasinensis* sp. nov. The innermost circle shows the GC content (the red line marks 50%) and the outermost circle shows the gene order, rRNAs, tRNAs, and PCGs. Plus (+) indicates the side of the major J-strand.

The mitochondrial genome of Symphyla until now was only known from two species: *Scutigerellacauseyae* and one undetermined species of *Symphylella* (Podsiadlowski et al. 2007; Gai et al. 2008). In the present study, we sequenced the complete mitogenome of *Scutigerellasinensis* sp. nov. The mitogenome of *S.sinensis* sp. nov. is 14 512 bp long and contains the control region (CR) and all 37 genes typically found in Arthropoda (Fig. 4, Table 3). The nucleotide composition varies along its length, being AT-rich for the entire mitogenome with: A – 36.41% (5284); T – 34.46% (5001); C – 19.41% (2817) and G – 9.71% (1410). With an AT content of 81% the AT rich or control region is 297 bp long and is located between trnQ and trnM. Three different start codons were present in the protein coding genes: ATG (6×) and ATA (2×), canonical codons encoding Methionine, and ATT (5×) encoding Isoleucine. Two different stop codons were present: TAA (11×) and TAG (2×). No truncated stop codon was observed (Table 3).

**Table 3.** Organization of *Scutigerellasinensis* sp. nov. mitochondrial genome.

Start	End	Length (bp)	Direction	Start/Stop codons	Gene name	Gene product
181	1303	1122	+	ATG/TAA	CYTB	Cytochrome C Oxidase 1
1307	1368	62	+		trnS2 (uga)	tRNA-Ser
1386	2286	901	-	ATA/TAA	ND1	NADH Dehydrogenase 1
2298	2360	63	-		trnL1 (uag)	tRNA-Leu
2360	2425	66	-		trnL2 (uaa)	tRNA-Leu
2371	3656	1286	-		l-rRNA	16S ribosomal RNA
3604	3665	62	-		trnV (uac)	tRNA-Val
3659	4408	750	-		s-rRNA	12S ribosomal RNA
4369	4425	57	-		trnQ (uug)	tRNA-Gln
4425	4722	297			CR	Control Region
4723	4786	64	+		trnM (cau)	tRNA-Met
4788	4851	64	+		trnI (aau)	tRNA-Ile
4881	5871	991	+	ATT/TAA	ND2	NADH Dehydrogenase 2
5876	5939	64	+		trnW (uca)	tRNA-Trp
5931	5986	56	-		trnC (gca)	tRNA-Cys
5985	6046	62	-		trnY (gua)	tRNA-Tyr
6038	7582	1545	+	ATA/TAA	COX1	Cytochrome C Oxidase I
7581	8256	676	+	ATG/TAA	COX2	Cytochrome C Oxidase II
8258	8321	64	+		trnK (cuu)	tRNA-Lys
8321	8387	67	+		trnD (guc)	tRNA-Asp
8387	8549	163	+	ATT/TAA	ATP8	ATP synthase F0 subunit 8
8542	9214	673	+	ATG/TAA	ATP6	ATP synthase F0 subunit 6
9213	10014	802	+	ATG/TAA	COX3	Cytochrome C Oxidase III
9997	10052	56	+		trnG (ucc)	tRNA-Gly
10052	10406	355	+	ATT/TAA	ND3	NADH Dehydrogenase 3
10412	10472	61	+		trnA (ugc)	tRNA-Ala
10472	10534	63	+		trnR (ucg)	tRNA-Arg
10537	10599	63	+		trnN (guu)	tRNA-Asn
10599	10653	55	+		trnS1 (gcu)	tRNA-Ser
10653	10715	63	+		trnE (uuc)	tRNA-Glu
10713	10769	57	-		trnF (gaa)	tRNA-Phe
10768	12436	1669	-	ATT/TAG	ND5	NADH Dehydrogenase 5
12445	12499	55	-		trnH (gug)	tRNA-His
12498	13821	1324	-	ATG/TAG	ND4	NADH Dehydrogenase 4
13814	14093	280	-	ATG/TAA	ND4L	NADH Dehydrogenase 4L
14095	14157	63	-		trnP (ugg)	tRNA-Pro
14158	14218	61	+		trnT (ugu)	tRNA-Thr
14217	181	477	+	ATT/TAA	ND6	NADH Dehydrogenase 6

**Figure 5.**
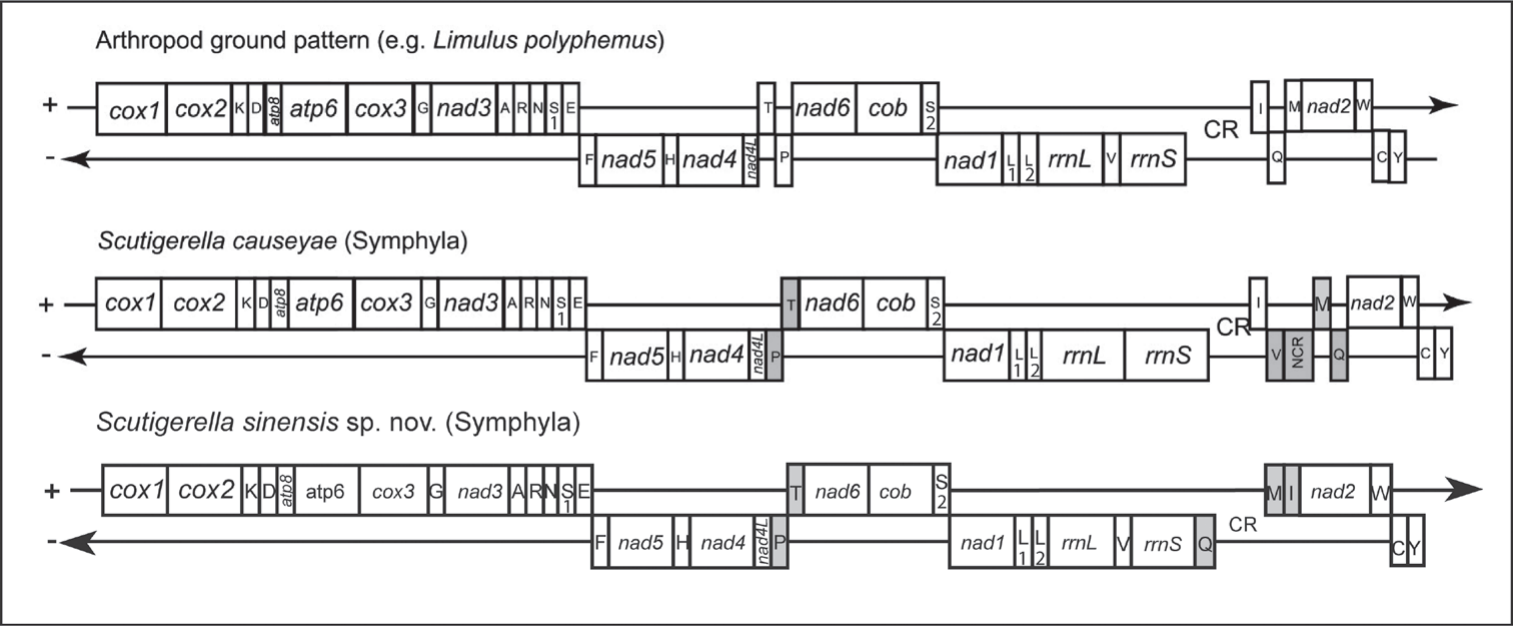
Mitochondrial gene arrangements of *Scutigerellasinensis* sp. nov. compared to *S.causeyae* and the arthropod ground pattern (adapted from Podsiadlowski et al. 2007). Genes shaded grey have different relative positions compared to the ground pattern. Upper line with (+)–strand genes, lower line with (–) –strand genes. CR: putative control region.

Compared to the mitogenome of the congeneric species *S.causeyae*, the new sequence is 125 bp smaller and differs in the relative position of three tRNA genes (Q, M, I) located next to the control region. Additionally, the tRNA-Valine is located between the rRNAs, like in the inferred arthropod ground pattern (Staton et al. 1997) (Fig. 5). This is the first report of the occurrence of this ground plan in *Scutigerella*, which is an important similarity between the new species and the hypothetical ancestor. On other hand, the translocation between the tRNA genes P and T is shared between *S.sinensis* and *S.causeyae*, but not observed in the ground pattern; it can be a step toward for further studies on character evolution in *Scutigerella*.

### ﻿COI-tree reconstruction

**Figure 6.**
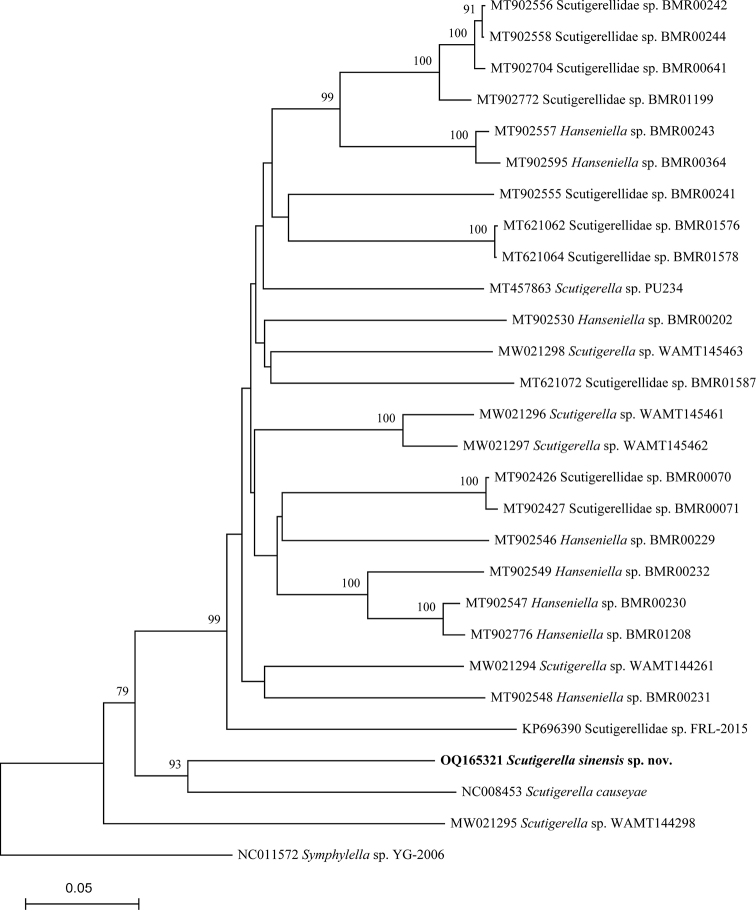
Neighbor-Joining tree (Jukes-Cantor model, Bootstrap 1000 replicates) of symphylans inferred from COI gene sequences. Numbers at the nodes show the bootstrap values > 50%.

In the Neighbor-Joining tree constructed based on DNA barcoding sequences, *S.sinensis* sp. nov. clustered with *S.causeyae* (Fig. 6). However, these two species very much differ in their morphological characters: the posterior margin of tergite 2 (deeply emarginated in *S.sinensis* sp. nov. vs. truncate and barely emarginated in *S.causeyae*), chaetotaxy on the head (a3 seta absent in *S.sinensis* sp. nov. vs. a3 seta present in *S.causeyae*) and shape of the stylus (both apical and subapical setae pointed in *S.sinensis* sp. nov. vs. apical seta truncate and subapical seta pointed in *S.causeyae*). The sister group of this cluster is a mixed assemblage of species determined as *Hanseniella*, *Scutigerella* and Scutigerellidae. Since most sequences downloaded from GenBank are from the individuals only primarily determined to family or genus levels and the validation of identification cannot be confirmed, we refrain from questioning the monophyletic status of *Hanseniella* and *Scutigerella*.

### ﻿Genetic divergence

The pairwise genetic distance of 28 sequences of symphylan species based on the K2P model is given in the Suppl. material 1. The genetic distance between *S.sinensis* sp. nov. and other congeners is 0.2747 on average (0.2280–0.2946), which gives further support for our morphological identification, however the coverage of species does not allow us to draw too many conclusions. The genetic distances of the COI gene among different taxonomic levels of Symphyla are given in Table 4 (but determinations of species downloaded from GenBank are questionable).

**Table 4.** Genetic distances of Symphyla analyzed by mitochondrial COI gene (K2P model).

Level	Mean	Minimum	Maximum
*Scutigerellasinensis* sp. nov. vs congeners	0.2747	0.2280	0.2946
Interspecific distances within the genus *Scutigerella*	0.2638	0.1883	0.3248
Interspecific distances within the genus *Hanseniella*	0.2084	0.1738	0.2343
Conspecific distances of the genus *Hanseniella*	0.0550	0.0160	0.0961
Intergeneric distances between *Scutigerella* and *Hanseniella*	0.2376	0.1700	0.3364
Interfamiliar distance between Scutigerellidae and Scolopendrellidae	0.3170	0.2947	0.3636

## Supplementary Material

XML Treatment for Scutigerella

XML Treatment for Scutigerella sinensis

## Supplementary materials

Supplementary material 1 K2P distances between different speices of Symphyla analyzed by the mitochondrial COI gene Data type table (excel file) https://binary.pensoft.net/file/833913This dataset is made available under the Open Database License (http://opendatacommons.org/licenses/odbl/1.0/). The Open Database License (ODbL) is a license agreement intended to allow users to freely share, modify, and use this Dataset while maintaining this same freedom for others, provided that the original source and author(s) are credited.Ya-Li Jin, Nerivania Nunes Godeiro, Yun Bu
